# PHYSICAL FUNCTION AND BODY COMPOSITION AS NUTRITIONAL OUTCOMES: ASSESSMENT APPROACHES

**DOI:** 10.1093/geroni/igz038.1443

**Published:** 2019-11-08

**Authors:** Connie W Bales, Kathryn N Porter Starr, Marshall Miller

**Affiliations:** Duke University, Durham, North Carolina, United States

## Abstract

Nutritional status is a strong determinant of both body composition and physical function (PF), parameters that are closely interrelated but rarely evaluated in the clinical setting due to cost, access, and lack of agreement on best approaches in older adults. Recent evidence that changes in muscle mass do not closely correspond to changes in muscle function will be reviewed in the context of our studies of higher protein obesity interventions. PF assessments, including indices for older adults (Short Physical Performance Battery and Physical Performance Test), as well as specific tests like gait speed and handgrip strength, will be explained as nutrition outcomes and in relation to body composition from air displacement (BodPod) and dual energy x-ray absorptiometry (DXA). These results, along with new studies of muscle quality, will bring a better understanding of the complexity of responses to nutritional interventions designed to optimize body mass and composition in older adults.

